# *N*-Alkyl-2-[4-(trifluoromethyl)benzoyl]hydrazine-1-carboxamides and Their Analogues: Synthesis and Multitarget Biological Activity

**DOI:** 10.3390/molecules25102268

**Published:** 2020-05-12

**Authors:** Martin Krátký, Zsuzsa Baranyai, Šárka Štěpánková, Katarína Svrčková, Markéta Švarcová, Jiřina Stolaříková, Lilla Horváth, Szilvia Bősze, Jarmila Vinšová

**Affiliations:** 1 Department of Organic and Bioorganic Chemistry, Faculty of Pharmacy in Hradec Králové, Charles University, Akademika Heyrovského 1203, 500 05 Hradec Králové, Czech Republic; komloova.m@seznam.cz (M.Š.); vinsova@faf.cuni.cz (J.V.); 2 MTA-ELTE Research Group of Peptide Chemistry, Hungarian Academy of Sciences, Eötvös Loránd University, Pázmány Péter Sétány 1/A, H-1117, 1518 Budapest, Hungary; baranyaizsuzs@gmail.com (Z.B.); lilla.horvath@yahoo.com (L.H.); szilvia.bosze@gmail.com (S.B.); 3 Department of Biological and Biochemical Sciences, Faculty of Chemical Technology, University of Pardubice, Studentská 573, 532 10 Pardubice, Czech Republic; sarka.stepankova@upce.cz (Š.Š.); katarina.svrckova@upce.cz (K.S.); 4 Department of Chemistry, Faculty of Science, J. E. Purkinje University, České mládeže 8, 400 96 Ústí nad Labem, Czech Republic; 5 Laboratory for Mycobacterial Diagnostics and Tuberculosis, Regional Institute of Public Health in Ostrava, Partyzánské námĕstí 7, 702 00 Ostrava, Czech Republic; jirina.stolarikova@zuova.cz

**Keywords:** antimycobacterial activity, acetylcholinesterase inhibition, butyrylcholinesterase inhibition, cytostatic properties, hydrazides, 4-(trifluoromethyl)benzohydrazide

## Abstract

Based on the isosterism concept, we have designed and synthesized homologous *N*-alkyl-2-[4-(trifluoromethyl)benzoyl]hydrazine-1-carboxamides (from C_1_ to C_18_) as potential antimicrobial agents and enzyme inhibitors. They were obtained from 4-(trifluoromethyl)benzohydrazide by three synthetic approaches and characterized by spectral methods. The derivatives were screened for their inhibition of acetylcholinesterase (AChE) and butyrylcholinesterase (BuChE) via Ellman’s method. All the hydrazinecarboxamides revealed a moderate inhibition of both AChE and BuChE, with IC_50_ values of 27.04–106.75 µM and 58.01–277.48 µM, respectively. Some compounds exhibited lower IC_50_ for AChE than the clinically used drug rivastigmine. *N*-Tridecyl/pentadecyl-2-[4-(trifluoromethyl)benzoyl]hydrazine-1-carboxamides were identified as the most potent and selective inhibitors of AChE. For inhibition of BuChE, alkyl chain lengths from C_5_ to C_7_ are optimal substituents. Based on molecular docking study, the compounds may work as non-covalent inhibitors that are placed in a close proximity to the active site triad. The compounds were evaluated against *Mycobacterium tuberculosis* H_37_Rv and nontuberculous mycobacteria (*M. avium*, *M. kansasii*). Reflecting these results, we prepared additional analogues of the most active carboxamide (*n*-hexyl derivative **2f**). *N*-Hexyl-5-[4-(trifluoromethyl)phenyl]-1,3,4-oxadiazol-2-amine (**4**) exhibited the lowest minimum inhibitory concentrations within this study (MIC ≥ 62.5 µM), however, this activity is mild. All the compounds avoided cytostatic properties on two eukaryotic cell lines (HepG2, MonoMac6).

## 1. Introduction

The development of novel drugs involves various medicinal chemistry approaches, including the widely used isostere/bioisostere strategy [1]. A bioisostere is a molecule that results from the exchange of an original atom or a group of atoms for an alternative, roughly similar atom or group of atoms. Based on physical and/or chemical similarity, isosteric compounds may share analogous pharmacological behavior. Usually, it is a way to ameliorate disadvantageous features of current drugs and drug candidates, e.g., low activity, drug resistance, toxicity, poor pharmacokinetic profile, etc. It is also possible to establish an original bioactivity [2,3].

**Scheme 1:**
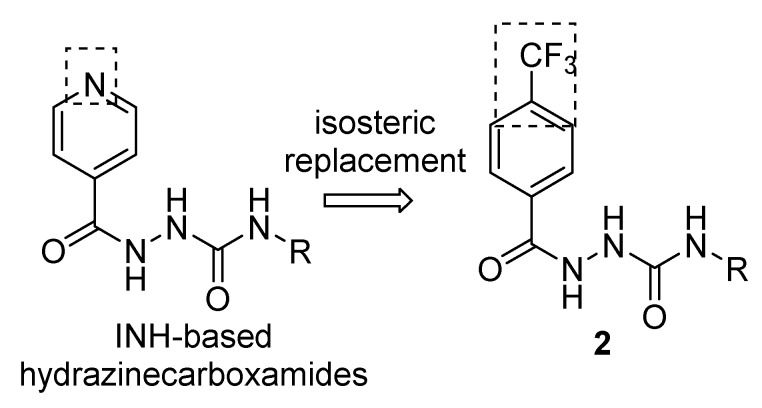
Design of the 1,2-diacylhydrazines **2** based on 4-(trifluoromethyl)hydrazide **1** scaffold.

In our previous study, we successfully implemented a bioisosteric concept for isoniazid-based hydrazones where the antimycobacterial drug isoniazid (isonicotinoylhydrazide, INH) was replaced by 4-(trifluoromethyl)benzohydrazide [4]. What is more, *N*^2^-substituted derivatives of 4-(trifluoro-methyl)benzohydrazide (**1**) have been reported as antibacterial molecules effective against both *Mycobacterium tuberculosis* and nontuberculous mycobacteria [4,5,6], Gram-positive cocci including methicillin-resistant *Staphylococcus aureus*, yeasts and molds [4], anticonvulsants [7,8], cytostatic/antiproliferative and cytotoxic [9,10], metal-chelating [5,10] or anti-inflammatory agents [9]. That is why we applied this approach of also for biologically active *N*-alkyl-2-isonicotinoylhydrazine-1-carboxamides [11,12] using replacement of INH scaffold by isosteric 4-(trifluoromethyl)hydrazide **1** (Scheme 1).

Then, we screened novel molecules for their antimicrobial and cytostatic properties. Moreover, since many scaffolds carrying aromatic trifluoromethyl group have showed inhibition of acetyl- and butyrylcholinesterase (AChE and BuChE) [13,14,15], we tested novel hydrazides also for this bioactivity.

## 2. Results and Discussion

### 2.1. Chemistry

**Scheme 2:**
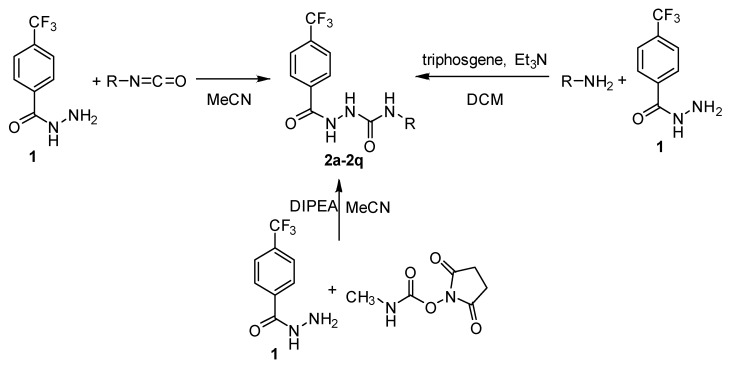
Synthesis of *N*-alkyl-2-[4-(trifluoromethyl)benzoyl]hydrazine-1-carboxamides **2a**–**2q** (R: *n*-alkyl from C_1_ to C_16_ and C_18_; DIPEA: *N*,*N*-diisopropylethylamine; DCM: dichloromethane).

Analogously to our previous works [11,12], hydrazine-1-carboxamides were obtained using three synthetic approaches. Most of the derivatives (**2b**–**2i**, **2k**, **2l**, **2n**, **2p**–**2q**) were prepared from commercially available isocyanates and the hydrazide **1** in acetonitrile. This reaction (method A) is simple, quick and it provides high yields, up to quantitative ones (89–99%). For the synthesis of *N*-decyl, tridecyl and pentadecyl derivatives **2i**, **2m** and **2o**, where the required isocyanates were not commercially available, we prepared them in situ using the corresponding amines and triphosgene in the presence of triethylamine (TEA) under a nitrogen atmosphere, followed by addition of the hydrazide **1**. The yields of this method B ranged from 78% to 91%. Finally, for the synthesis of *N*-methyl derivative **1a**, *N*-succinimidyl *N*-methylcarbamate in the presence of a non-nucleophilic tertiary base (*N*,*N*-diisopropylethylamine, DIPEA) was used as a non-toxic and crystalline methyl isocyanate substitute (method C with a good yield of 77%). An overview of the synthetic approaches used is depicted in Scheme 2.

**Scheme 3:**
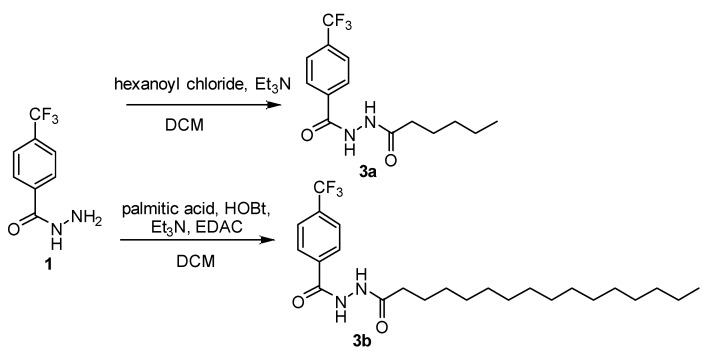
Synthesis of 1,2-diacylhydrazines **3** (DCM: dichloromethane; HOBt: 1-hydroxybenzotriazole; EDAC: 1-ethyl-3-(3-dimethylaminopropyl)carbodiimide hydrochloride).

Based on the antimycobacterial activity of the derivative **2f**, we decided to prepare several its analogues. First, we investigated the 1,2-diacylhydrazines **3** (Scheme 3), i.e., derivative with no secondary amine group. The *N*´-hexyl derivative **3a** was prepared via direct acylation from the **1** and hexanoyl chloride in the presence of base (TEA; yield 79%). In order to investigate the length of the acyl chain, also *N*´-hexadecanoyl derivative **3b** was synthesized using carbodiimide (EDAC)/1-hydroxybenzotriazole (HOBt)-mediated coupling (97%).

**Scheme 4:**
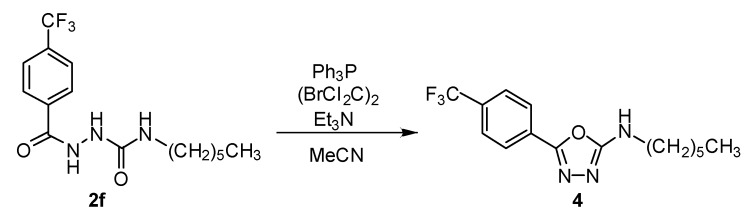
Synthesis of 1,3,4-oxadiazole-2-amine **4** (Ph_3_P: triphenylphosphine; (BrCl_2_C)_2_: 1,2-dibromo-1,1,2,2-tetrachloroethane).

According to [11], the derivative **2f** was cyclized to corresponding *N*-hexyl-5-[4-(trifluoromethyl)phenyl]-1,3,4-oxadiazol-2-amine (**4**) by treatment with triphenylphosphine, 1,2-dibromo-1,1,2,2-tetrachloroethane and TEA in anhydrous acetonitrile (82%; Scheme 4).

All the compounds **2**–**4** (Table 1) were characterized by their ^1^H- and ^13^C-NMR and infrared spectra and melting points. Additionally, the purity was checked by thin-layer chromatography (TLC) and elemental analysis.

### 2.2. Inhibition of Acetyl- and Butyrylcholinesterase

**Figure 1:**
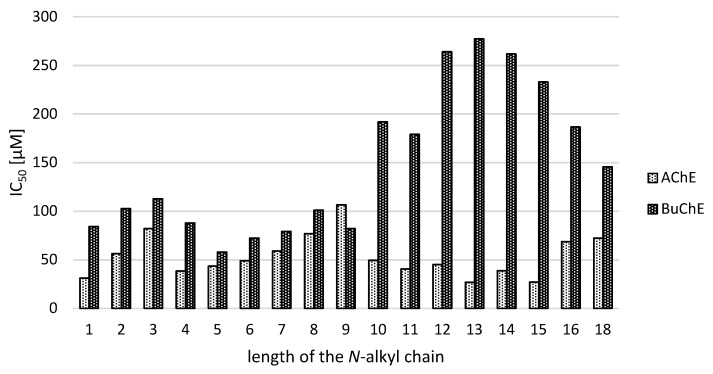
The dependence of enzyme inhibition on alkyl chain length of *N*-substituted-2-[4-(trifluoromethyl)benzoyl]hydrazine-1-carboxamide scaffold **2**.

Newly synthesized *N*-alkyl-2-[4-(trifluoromethyl)benzoyl]hydrazine-1-carboxamides **2a**–**2q** were evaluated for their in vitro potency to inhibit AChE from electric eel (*Ee*AChE) and BuChE from equine serum (EqBuChE) using modified Ellman’s spectrophotometric method [16]. The results (Table 1 and Figure 1) were compared with those determined for rivastigmine, a clinically used drug for treatment of various dementia. From chemical point of view, it is an aromatic carbamate-based dual inhibitor of both cholinesterases. The efficacy of inhibitors is expressed as IC_50_, i.e., the concentration causing 50% inhibition of the enzyme activity. Based on IC_50_ values for both enzymes, selectivity indexes (SI) as the ratio of IC_50_ for BuChE/IC_50_ for AChE to quantify the preference for AChE were calculated (Table 1). The results were compared with those obtained for rivastigmine, an established carbamate used in the therapy of not only Alzheimer’s and Parkinson’s dementias due to its cholinergic effect. From molecular pharmacology point of view, this drug belongs to dual acylating pseudo-irreversible inhibitors of both AChE and BuChE.

Generally, with only one exception (the nonyl derivative **2i**), the hydrazine-1-carboxamides **2** are stronger inhibitors of AChE. Focusing on this enzyme, IC_50_ values were found in a close range of 27.04 (**2o**)–106.75 (**2i**) µM. The values of ten compounds (**2a**, **2d**–**2f**, **2j**–**2o**) are superior to the IC_50_ for rivastigmine (56.10 µM), the other two (**2b** and **2g**) produced comparable in vitro activity. By comparison with the parent hydrazide **1**, the majority of its carboxamides (**2a**, **2b**, **2d**–**2g**, and **2j**–**2o**) showed an improved inhibition of AChE; thus, this structural modification can be considered as successful increasing the activity by up to 2.6 times (**1** vs. **2o** and **2m**).

Of course, in these analogues the length of *N*-alkyl chain is the only structural factor influencing the inhibitory properties. Interestingly, the elongation of the substituent decreases the activity (from C_1_ to C_3_, 31.2 and 82.3 µM, respectively). Then a drop of IC_50_ value was observed (IC_50_ of C_4_ = 38.6 µM) followed by analogous gradual reduction of the inhibitory properties up to the global minimum of 106.8 µM (**2i**). Next three compounds (decyl, undecyl and dodecyl **2j**–**2l**) exhibited similar improved activity around 40–50 µM, followed by even more efficient tetradecyl (**2n**) and especially the best tridecyl **2m** and pentadecyl **2o** derivatives (IC_50_ 28.9 and 27.0 µM, respectively). Then, IC_50_ values are increasing slowly again (up to C_18_ with 72.31 µM). Starting from C_10_ to C_16_, even-odd effect can be observed favoring odd alkyls. These structure-activity relationships and trends are depicted in Figure 1.

Analyzing results of BuChE inhibition, somewhat different results and structure-activity relationships were found. Overall, IC_50_ values for BuChE were higher and in a broader concentration range from 58.0 µM (*N*-pentyl molecule **2e**) up to 277.5 µM (the most active AChE inhibitor **2m**). None of the derivatives **2** exhibited more potent inhibition than rivastigmine (38.4 µM). Notably, twelve carboxamides showed an improved inhibition of the parent 4-CF_3_-benzohydrazide **1** (**2a**–**2k**, **2p**, **2q**), even up to 3.5 times (**1** vs. **2e**). Clearly, *N*-*n*-alkyl from butyl to nonyl (**2d**–**2i**) is essential for enhanced BuChE inhibition with an optimum of 5–7 carbons (Figure 1). The gradual diminishing of the enzyme inhibition potency was observed for these extending chain length: from C_1_ to C_3_, C_5_ → C_8_, C_9_ → C_10_, C_11_ → C_13_.

Analogous “oscillating” activity without one dominant trend depending on alkyl chain length has been described previously, e.g., by Imramovský et al. [17]. There are many factors influencing inhibition of AChE and BuChE, not solely the length of *n*-alkyl chain, e.g., such as lipophilicity, electronic and steric effects. Moreover, inhibitors of AChE can interact with more enzyme “subsites”, either with one or with more at once. They may interfere with peripheral anionic site, catalytic esteratic subsite (competitively, irreversibly or pseudo-irreversibly), anionic site in the active site, or they can bind into a narrow aromatic gorge, thus preventing access of the substrate to the catalytic triad [18]. Based on the alkyl length, a change of fitting into enzyme can occur. The alkyls that are flexible may also adopt a conformation that allows a better interaction with the enzyme.

All the derivatives **2** act as dual inhibitors of both cholinesterase enzymes. With an exception of *N*-nonyl carboxamide **2i**, the derivatives produced more intense inhibition of AChE. We used selectivity index for its quantification. Some of short and intermediate alkyls led to a comparatively balanced inhibition of both cholinesterases (SI ≤ 1.5; propyl **2c**, from pentyl **2e** to octyl **2h**). Contrarily, four derivatives led to a significantly preferential inhibition of AChE (SI > 5; **2l**–**2o**). Notably, an escalated AChE selectivity is related to longer alkyls (from C_10_ to C_15_).

Then, we screened an inhibition of cholinesterases caused by three analogues synthesized for their potential antimycobacterial activity primarily (**3a**, **3b** and **4**). These derivatives produced identical or decreased inhibition of AChE when compared to the parent hydrazide **1** (67.1–91.9 µM vs. 69.4 µM). On the other hand, all of them are more potent inhibitors of BuChE (IC_50_ values from 110 to 148.6 µM) with similar selectivity indexes. The shorter carbon chain (C_6_) is preferred over long one (C_16_). In general, the amides **3** and the oxadiazole **4** do not overcome the in vitro effect of the hydrazinecarboxamides **2**.

#### Molecular Docking Study

In order to presume possible binding mode of the prepared molecules with human AChE (pdb code 4PQE) and BChE (pdb code 1POI), molecular modelling study was performed. The most potent compound **2o** and the third most active derivative **2a** against AChE, were chosen as representatives for thorough investigation of ligand-enzyme interactions in the active site of AChE. Similarly, the carboxamide **2e**, being the most potent molecule against BuChE, was used for the determination of the binding mode in BuChE.

**Figure 2:**
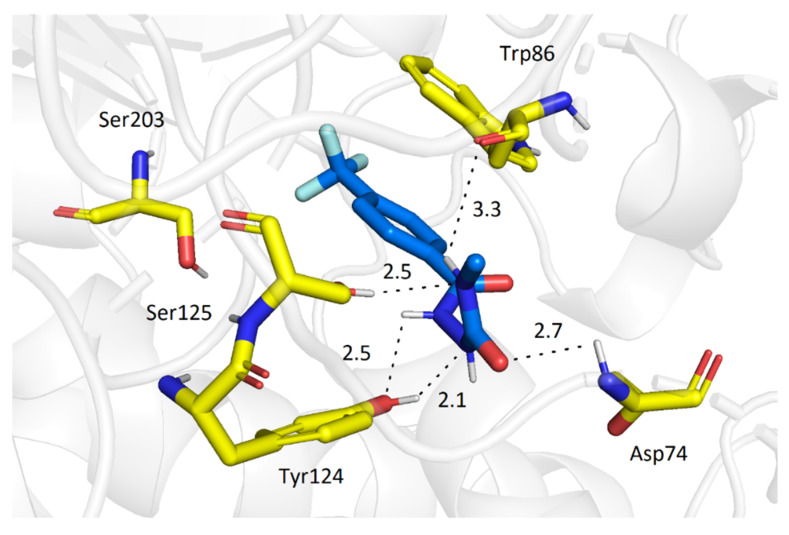
Molecular interactions of **2a** (blue) and AChE.

**Figure 3:**
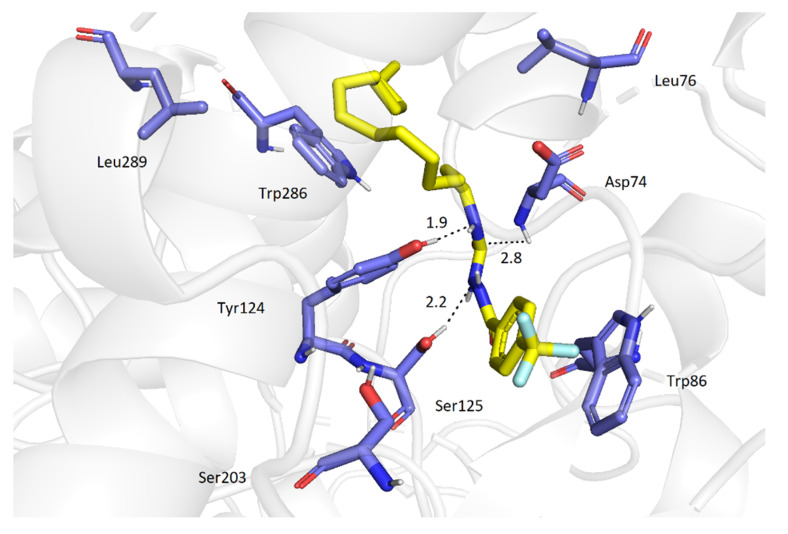
Molecular interactions of **2o** (yellow) and AChE.

The best docking pose of **2a** in AChE showed the molecule of the ligand placed deep within the cavity, in a close proximity to the active site triad (Figure 2). A significant amount of H-bonds (with Ser125, Tyr124, Trp86 and Asp74) indicates highly favorable orientation of **2a**. Additionally, π-π stacking with Trp86 further stabilizes the ligand-enzyme non-covalent complex. Also, the carboxamide **2o** binds closely to the catalytic triad at the bottom of the cavity (Figure 3) in a similar manner. There are three hydrogen bonds (with Ser125, Tyr124 and Asp74) and, in addition, this binding mode is stabilized by π-π stacking interaction with Trp86. The long tridecyl chain is heading out of the gorge (forming hydrophobic interactions with Leu76, Leu289 and Trp286), thereby hindering access of AChE to the active site.

**Figure 4:**
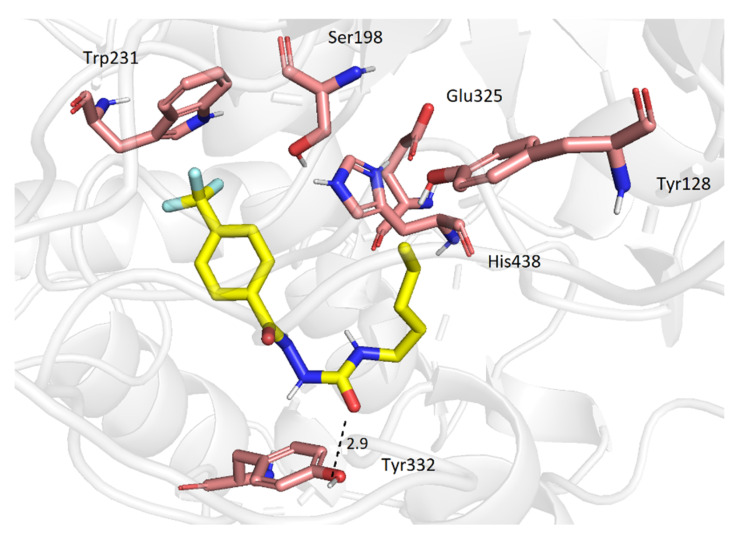
Molecular interactions of **2e** (yellow) and BuChE.

Concerning BuChE, all the presented compounds displayed a similar orientation in the active site of BuChE, regardless of the growing size of their molecules. This may be due to a relatively spacious cavity of BuChE compared to the one in AChE. However, the derivative **2e** displayed only a few detectable interactions, namely H-bond with Tyr332 and CF-π with Trp231 (Figure 4). Nevertheless, the ligand possesses the ability to block the catalytic triad completely.

### 2.3. Antimycobacterial Activity

Since the hydrazine-1-carboxamides **2** are isosteres of previously reported antimycobacterial 2-isonicotinoylhydrazine-1-carboxamides [11], initially we screened in vitro antimycobacterial activity of the derivatives **2**. The panel of mycobacteria covers drug-susceptible strain *Mycobacterium tuberculosis* 331/88 (i.e., H_37_Rv) and three atypical mycobacterial strains, namely *Mycobacterium avium* 330/88 (polydrug-resistant) and one collection strains of *Mycobacterium kansasii* (235/80) and a clinical isolate (*M. kansasii* 6509/96).

For most compounds, the exact determination of minimum inhibitory concentrations (MIC) was not feasible due to their limited solubility in the testing medium (evidenced by precipitation and/or turbidity); their MIC exceeded 250 µM. None of the derivatives **1**–**4** was capable of *M. avium* inhibition. *M. tuberculosis* and both strains of *M. kansasii* were inhibited by six compounds. Among primarily designed derivatives **2**, the derivatives with the shortest alkyl chains (**2a** and **2b**) were negligibly active (MIC of 1000 µM). On the other hand, *N*-hexyl derivative **2f** exhibited a uniform MIC of 250 µM. Thus, we synthesized and evaluated its analogues **3** and **4**. The cyclization of the carboxamide **2f** to 1,3,4-oxadiazole **4** retained potency for *M. tuberculosis* (MIC of 125–250 µM) and improved activity against *M. kansasii* mildly (62.5–250 µM). *N*´-hexanoylhydrazide **3a** exhibited lower MIC for *M. tuberculosis* (125 µM), but this modification hampered efficacy against *M. kansasii* (>250 µM).

Drawing a comparison between the parent hydrazide **1** [4] and its analogues, two derivatives were superior (**3a**, **4**) and the activity of **2f** was identical. All of these compounds produced lower MIC against *M. kansasii*. The first-line antituberculotic hydrazide drug isoniazid (INH), involved for comparison, exhibited significantly higher growth inhibition of *M. tuberculosis* (1 µM) and the isolate of *M. kansasii* from a patient. On the other hand, the hydrazide **2f** and the oxadiazole **4** were superior against *M. kansasii* 235/80.

Despite these structure-activity relationships described, the activity against both tuberculous and nontuberculous mycobacteria is significantly lower than in the case of original *N*-alkyl-2-isonicotinoylhydrazine-1-carboxamides, i.e., derivatives of INH. That is why no MIC for multidrug-resistant *M. tuberculosis* strains were determined.

### 2.4. Cytostatic Properties

Since compounds containing trifluoromethyl group has been known for their toxic action on eukaryotic cells and approved as anticancer drugs [19], we investigated cytostatic properties of the derivatives 2–4 using human hepatocellular carcinoma (HepG2) and monocyte (MonoMac6) cell lines. The compounds with shorter alkyls (**2a**–**2d**) and parent 4-(trifluoromethyl)benzohydrazide (**1**) as well avoided any cytostatic action for both cell lines at a concentration of 100 µM. The remaining compounds have no cytostatic effect up to 50 µM. Higher concentrations were not investigated due to solubility problems, i.e., precipitation of crystals. In sum, none of the compounds showed any cytostatic properties against these eukaryotic cells.

## 3. Materials and Methods

### 3.1. Chemistry

#### 3.1.1. General

All the reagents and solvents were purchased from Sigma-Aldrich (Darmstadt, Germany) or Penta Chemicals (Prague, Czech Republic) and they were used as received. The purity of the compounds was monitored by thin-layer chromatography (TLC). TLC plates were coated with 0.2 mm Merck 60 F254 silica gel (Merck Millipore, Darmstadt, Germany) with UV detection (254 nm). The melting points were determined on a B-540 Melting Point apparatus (Büchi, Flawil, Switzerland) using open capillaries and they are uncorrected. Infrared spectra were recorded on a FT-IR spectrometer using the ATR-Ge method (Nicolet 6700 FT-IR, Thermo Fisher Scientific, Waltham, MA, USA) in the range of 650–4000 cm^−1^. The NMR spectra were measured in DMSO-*d*_6_ or *N*,*N*-dimethylformamide-*d*_7_ (DMF-*d*_7_) at ambient and higher (80 °C) temperature using a Varian V NMR S500 instrument (500 MHz for ^1^H and 126 MHz for ^13^C; Varian Corp., Palo Alto, CA, USA). The chemical shifts δ are given in ppm and were referred indirectly to tetramethylsilane via signals of DMSO-*d*_6_ (2.50 for ^1^H and 39.51 for ^13^C spectra) or DMF-*d*_7_ (2.75, 2.92 and 8.03 for ^1^H, 29.76, 34.89 and 163.15 for ^13^C spectra). The coupling constants (*J*) are reported in Hz. Elemental analysis was performed on a Vario MICRO Cube Element Analyzer (Elementar Analysensysteme, Hanau, Germany). Both calculated and found values are given as percentages.

The calculated log*P* values (Clog*P*) that are the logarithms of the partition coefficients for octan-1-ol/water, were determined using the program CS ChemOffice Ultra version 18.0 (CambridgeSoft, Cambridge, MA, USA).

The identity of the known compounds was established using NMR and IR spectroscopy. Additionally, their purity was checked by melting points measurement and elemental analysis. The compounds were considered pure if they agree within ±0.4% with theoretical values.

#### 3.1.2. Synthesis of *N*-alkyl Hydrazine-1-carboxamides **2**

##### Method A

4-(Trifluoromethyl)benzohydrazide (**1**, 204.2 mg, 1.0 mmol) was dissolved in anhydrous acetonitrile (MeCN, 8 mL) and then the appropriate isocyanate (1.05 mmol) was added in one portion. The reaction mixture was stirred at the room temperature for 8 h, then stored for 2 h at −20 °C. Resulting precipitate was filtered off, washed with a small volume of MeCN and dried. The products were recrystallised from ethyl acetate if necessary.

##### Method B

Method B is based on generation of an appropriate isocyanate in situ. Triphosgene (bis(trichloromethyl)carbonate; 118.7 mg, 0.4 mmol) was dissolved in anhydrous dichloromethane (DCM; 5 mL) under nitrogen atmosphere and the appropriate amine (1.01 mmol) dissolved in anhydrous DCM (5 mL) was added dropwise. The mixture was stirred for 30 min at room temperature, then treated with triethylamine (TEA; 293 µL, 2.1 mmol). After 30 min, 4-(trifluoromethyl)benzohydrazide (**1**, 204.2 mg, 1.0 mmol) was added. The reaction mixture was stirred for 10 h at room temperature, then evaporated to dryness, treated with water (10 mL) and extracted with ethyl acetate (3 × 15 mL). The combined organic phase was dried over anhydrous sodium sulphate, filtered off and evaporated to dryness to give the final product, which was crystallised from ethyl acetate.

##### Method C

4-(Trifluoromethyl)benzohydrazide (**1**, 204.2 mg, 1.0 mmol) was dissolved in acetonitrile (5 mL) and mixed with *N*,*N*-diisopropylethylamine (DIPEA; 2.0 mmol, 348 µL) and *N*-succinimidyl *N*-methylcarbamate (258.2 mg, 1.5 mmol). The reaction mixture was stirred at the room temperature for 24 h, formed precipitate was filtered off and crystallised from ethyl acetate.

#### 3.1.3. Synthesis of 1,2-diacylhydrazines **3**

##### Method A

4-(Trifluoromethyl)benzohydrazide (**1**, 204.2 mg, 1.0 mmol) was dissolved in DCM (8 mL) together with triethylamine (1.5 mmol, 209 µL). Then, an acyl chloride (1.1 mmol) was added in one portion. The reaction mixture was stirred at the room temperature for 1 h. Then, resulted precipitate was filtered off, washed with a small volume of DCM and crystallised from ethyl acetate.

##### Method B

4-(Trifluoromethyl)benzohydrazide (**1**, 204.2 mg, 1.0 mmol) was mixed with triethylamine (1.5 mmol, 209 µL), 1-hydroxybenzotriazole hydrate (HOBt; 1 mmol, 153.1 mg) and appropriate acid (1 mmol) in DCM (8 mL). The mixture was cooled down to 0 °C. Then, 1-ethyl-3-(3-dimethylaminopropyl)carbodiimide hydrochloride (EDAC; 1.5 mmol, 287.6 mg) was added in one portion and the reaction mixture was let stir at the room temperature. After 48 h, resulted precipitate was filtered off, washed with a small volume of DCM and crystallised from ethyl acetate.

#### 3.1.4. Synthesis of 1,3,4-oxadiazole **4**

Triphenylphosphine (1.5 mmol, 393.4 mg) was added to the stirred suspension of 1,2-dibromo-1,1,2,2-tetrachloroethane (0.83 mmol, 271.1 mg) and *N*-hexyl-2-[4-(trifluoromethyl)benzoyl]-hydrazine-1-carboxamide (**2f**, 0.75 mmol, 248.5 mg) in anhydrous MeCN (4 mL) at room temperature. The reaction mixture was stirred at room temperature for 10 min and then cooled to 0 °C. Triethylamine (3.3 mmol, 459 µL) was added dropwise, and the stirring continued for an additional 10 h. Resulting crystals were filtered off, washed with a small volume of water followed by MeCN and dried to provide pure **4**.

### 3.2. Product Characterization

*N-Methyl-2-[4-(trifluoromethyl)benzoyl]hydrazine-1-carboxamide* (**2a**). White solid; yield 77% (method C); mp 237–238.5 °C. IR (ATR): 3310, 3269, 3074, 1656, 1643, 1583, 1546, 1509, 1425, 1411, 1326, 1315, 1260, 1165, 1116, 1071, 1016, 914, 858, 766, 694, 643, 629 cm^−1^. ^1^H-NMR (DMSO-*d*_6_): δ 10.30 (1H, s, NH), 8.05 (2H, d, *J* = 8.1 Hz, H2, H6), 7.93 (1H, s, NH), 7.83 (2H, d, *J* = 8.1 Hz, H3, H5), 6.44 (1H, q, *J* = 6.1 Hz, NH-alkyl), 2.54 (3H, d, *J* = 6.1 Hz, CH_3_). ^13^C-NMR (DMSO-*d*_6_): δ 165.49, 158.93, 136.83, 132.00 (q, *J* = 31.9 Hz), 128.71, 125.61 (q, *J* = 3.8 Hz), 124.41 (q, *J* = 272.5 Hz), 26.76. Anal. Calcd for C_10_H_10_F_3_N_3_O_2_ (261.20): C, 45.98; H, 3.86; N, 16.09. Found: C, 45.79; H, 4.01; N, 15.93.

*N-Ethyl-2-[4-(trifluoromethyl)benzoyl]hydrazine-1-carboxamide* (**2b**). White solid; yield 89% (method A); mp 231.5–232.5 °C. IR (ATR): 3309, 3071, 2984, 1666, 1640, 1576, 1545, 1508, 1461, 1379, 1325, 1313, 1255, 1173, 1120, 1085, 1069, 1015, 858, 694, 625 cm^−1^. ^1^H-NMR (DMSO-*d*_6_): δ 10.32 (1H, s, NH), 8.07 (2H, d, *J* = 8.0 Hz, H2, H6), 7.90 (1H, s, NH), 7.86 (2H, d, *J* = 8.1 Hz, H3, H5), 6.53 (1H, t, *J* = 5.7 Hz, NH-alkyl), 3.09–3.02 (2H, m, CH_2_), 1.01 (3H, t, *J* = 7.1 Hz, CH_3_). ^13^C-NMR (DMSO-*d*_6_): δ 165.40, 158.21, 136.81, 131.65 (q, *J* = 31.9 Hz), 128.66, 125.47 (q, *J* = 3.7 Hz), 124.08 (q, *J* = 272.7 Hz), 34.24, 15.70. Anal. Calcd for C_11_H_12_F_3_N_3_O_2_ (275.23): C, 48.00; H, 4.39; N, 15.27. Found: C, 48.15; H, 4.44; N, 14.98.

*N-Propyl-2-[4-(trifluoromethyl)benzoyl]hydrazine-1-carboxamide* (**2c**). White solid; yield 97% (method A); mp 227.5–228.5 °C. IR (ATR): 3273, 2967, 2878, 1688, 1657, 1647, 1558, 1543, 1489, 1328, 1262, 1170, 1128, 1071, 1017, 901, 859, 776, 631 cm^−1^. ^1^H-NMR (DMSO-*d*_6_): δ 10.32 (1H, s, NH), 8.07 (2H, d, *J* = 8.1 Hz, H2, H6), 7.89–7.84 (3H, m, NH, H3, H5), 6.53 (1H, t, *J* = 6.0 Hz, NH-alkyl), 2.98 (2H, dt, *J* = 7.4, 6.1 Hz, N-CH_2_), 1.40 (2H, sext, *J* = 7.3 Hz, CH_2_), 0.82 (3H, t, *J* = 7.4 Hz, CH_3_). ^13^C-NMR (DMSO-*d*_6_): δ 165.38, 158.34, 136.81, 131.63 (q, *J* = 31.9 Hz), 128.65, 125.48 (q, *J* = 3.8 Hz), 124.08 (q, *J* = 272.4 Hz), 41.18, 23.21, 11.41. Anal. Calcd for C_12_H_14_F_3_N_3_O_2_ (289.25): C, 49.83; H, 4.88; N, 14.53. Found: C, 50.05; H, 4.64; N, 14.80.

*N-Butyl-2-[4-(trifluoromethyl)benzoyl]hydrazine-1-carboxamide* (**2d**). White solid; yield 96 % (method A); mp 224.5–225.5 °C. IR (ATR): 3295, 2967, 2938, 2879, 1662, 1642, 1581, 1543, 1508, 1489, 1340, 1326, 1315, 1245, 1170, 1121, 1072, 1015, 857, 694, 636 cm^−1^. ^1^H-NMR (DMSO-*d*_6_): δ 10.32 (1H, s, NH), 8.07 (2H, d, *J* = 8.1 Hz, H2, H6), 7.89–7.84 (3H, m, NH, H3, H5), 6.51 (1H, t, *J* = 5.8 Hz, NH-alkyl), 3.02 (2H, q, *J* = 6.6 Hz, N-CH_2_), 1.37 (2H, p, *J* = 7.3 Hz, C^2^H_2_), 1.26 (2H, sext, *J* = 7.3 Hz, C^3^H_2_), 0.86 (3H, t, *J* = 7.3 Hz, CH_3_). ^13^C-NMR (DMSO-*d*_6_): δ 165.38, 158.32, 136.81, 131.64 (q, *J* = 31.9 Hz), 128.65, 125.47 (q, *J* = 3.8 Hz), 124.07 (q, *J* = 272.4 Hz), 39.05, 32.15, 19.59, 13.87. Anal. Calcd for C_13_H_16_F_3_N_3_O_2_ (303.28): C, 51.48; H, 5.32; N, 13.86. Found: C, 51.33; H, 5.60; N, 13.78.

*N-Pentyl-2-[4-(trifluoromethyl)benzoyl]hydrazine-1-carboxamide* (**2e**). White solid; yield 98 % (method A); mp 227–228 °C. IR (ATR): 3297, 3074, 2936, 2877, 1655, 1639, 1582, 1547, 1510, 1481, 1333, 1326, 1315, 1258, 1238, 1171, 1122, 1071, 1015, 858, 694, 642, 629 cm^−1^. ^1^H-NMR (DMSO-*d*_6_): δ 10.31 (1H, s, NH), 8.07 (2H, d, *J* = 8.2 Hz, H2, H6), 7.88–7.84 (3H, m, NH, H3, H5), 6.51 (1H, t, *J* = 5.8 Hz, NH-alkyl), 3.01 (2H, q, *J* = 6.6 Hz, N-CH_2_), 1.39 (2H, p, *J* = 7.2 Hz, C^2^H_2_), 1.31–1.18 (4H, m, C^3^H_2_, C^4^H_2_), 0.85 (3H, t, *J* = 7.0 Hz, CH_3_). ^13^C-NMR (DMSO-*d*_6_): δ 165.37, 158.30, 136.80, 131.63 (q, *J* = 31.9 Hz), 128.64, 125.46 (q, *J* = 3.7 Hz), 124.07 (q, *J* = 272.5 Hz), 39.36, 29.68, 28.67, 22.05, 14.09. Anal. Calcd for C_14_H_18_F_3_N_3_O_2_ (317.31): C, 52.99; H, 5.72; N, 13.24. Found: C, 52.78; H, 5.68; N, 13.36.

*N-Hexyl-2-[4-(trifluoromethyl)benzoyl]hydrazine-1-carboxamide* (**2f**). White solid; yield 99 % (method A); mp 209.5–210 °C. IR (ATR): 3296, 2930, 2862, 1668, 1640, 1580, 1543, 1508, 1471, 1343, 1326, 1314, 1268, 1254, 1171, 1120, 1072, 1015, 857, 719, 696, 627 cm^−1^. ^1^H-NMR (DMSO-*d*_6_): δ 10.28 (1H, s, NH), 8.04 (2H, d, *J* = 8.1 Hz, H2, H6), 7.85–7.82 (3H, m, NH, H3, H5), 6.48 (1H, t, *J* = 5.8 Hz, NH-alkyl), 2.97 (2H, q, *J* = 6.5 Hz, N-CH_2_), 1.35 (2H, p, *J* = 7.3 Hz, C^2^H_2_), 1.26–1.18 (6H, m, C^3^H_2_, C^4^H_2_, C^5^H_2_), 0.82 (3H, t, *J* = 7.0 Hz, CH_3_). ^13^C-NMR (DMSO-*d*_6_): δ 165.74, 158.67, 137.18, 131.99 (q, *J* = 31.9 Hz), 129.01, 125.85 (q, *J* = 3.8 Hz), 124.44 (q, *J* = 272.6 Hz), 39.78, 31.58, 30.34, 26.50, 22.62, 14.44. Anal. Calcd for C_15_H_20_F_3_N_3_O_2_ (331.33): C, 54.37; H, 6.08; N, 12.68. Found: C, 54.55; H, 6.00; N, 12.49.

*N-Heptyl-2-[4-(trifluoromethyl)benzoyl]hydrazine-1-carboxamide* (**2g**). White solid; yield 95 % (method A); mp 210.5–212.5 °C. IR (ATR): 3303, 3072, 2955, 2927, 2857, 1668, 1638, 1582, 1548, 1471, 1379, 1327, 1311, 1258, 1171, 1120, 1072, 1015, 856, 696, 629 cm^−1^. ^1^H-NMR (DMSO-*d*_6_): δ 10.31 (1H, s, NH), 8.07 (2H, d, *J* = 8.1 Hz, H2, H6), 7.88–7.84 (3H, m, NH, H3, H5), 6.51 (1H, t, *J* = 5.8 Hz, NH-alkyl), 3.00 (2H, q, *J* = 6.6 Hz, N-CH_2_), 1.38 (2H, p, *J* = 7.0 Hz, C^2^H_2_), 1.29–1.18 (8H, m, C^3^H_2_, C^4^H_2_, C^5^H_2_, C^6^H_2_), 0.84 (3H, t, *J* = 6.8 Hz, CH_3_). ^13^C-NMR (DMSO-*d*_6_): δ 165.36, 158.31, 136.80, 131.63 (q, *J* = 31.9 Hz), 128.64, 125.45 (q, *J* = 3.7 Hz), 124.06 (q, *J* = 272.4 Hz), 39.38, 31.45, 30.01, 28.64, 26.42, 22.21, 14.09. Anal. Calcd for C_16_H_22_F_3_N_3_O_2_ (345.36): C, 55.64; H, 6.42; N, 12.17. Found: C, 55.59; H, 6.62; N, 12.32.

*N-Octyl-2-[4-(trifluoromethyl)benzoyl]hydrazine-1-carboxamide* (**2h**). White solid; yield 98 % (method A); mp 217–218 °C. IR (ATR): 3295, 2926, 2856, 1654, 1639, 1581, 1546, 1481, 1327, 1315, 1249, 1171, 1121, 1073, 1015, 858, 694, 632 cm^−1^. ^1^H-NMR (DMSO-*d*_6_): δ 10.32 (1H, s, NH), 8.08 (2H, d, *J* = 8.1 Hz, H2, H6), 7.89–7.84 (3H, m, NH, H3, H5), 6.51 (1H, t, *J* = 5.9 Hz, NH-alkyl), 3.01 (2H, q, *J* = 6.6 Hz, N-CH_2_), 1.38 (2H, p, *J* = 6.7 Hz, C^2^H_2_), 1.29–1.18 (10H, m, C^3^H_2_, C^4^H_2_, C^5^H_2_, C^6^H_2_, C^7^H_2_), 0.84 (3H, t, *J* = 6.7 Hz, CH_3_). ^13^C-NMR (DMSO-*d*_6_): δ 165.37, 158.32, 136.81, 131.64 (q, *J* = 32.0 Hz), 128.64, 125.45 (q, *J* = 3.8 Hz), 124.07 (q, *J* = 272.5 Hz), 39.40, 31.42, 30.02, 28.96, 28.88, 26.48, 22.26, 14.09. Anal. Calcd for C_17_H_24_F_3_N_3_O_2_ (359.39): C, 56.81; H, 6.73; N, 11.69. Found: C, 56.68; H, 6.69; N, 11.53.

*N-Nonyl-2-[4-(trifluoromethyl)benzoyl]hydrazine-1-carboxamide* (**2i**). White solid; yield 99 % (method A); mp 204–206 °C. IR (ATR): 3297, 3071, 2925, 2854, 1666, 1638, 1582, 1546, 1471, 1343, 1327, 1315, 1257, 1170, 1121, 1072, 1015, 857, 695, 628 cm^−1^. ^1^H-NMR (DMSO-*d*_6_): δ 10.32 (1H, s, NH), 8.07 (2H, d, *J* = 8.1 Hz, H2, H6), 7.88–7.83 (3H, m, NH, H3, H5), 6.51 (1H, t, *J* = 5.7 Hz, NH-alkyl), 3.00 (2H, q, *J* = 6.6 Hz, N-CH_2_), 1.37 (2H, p, *J* = 6.9 Hz, C^2^H_2_), 1.28–1.18 (12H, m, C^3^H_2_, C^4^H_2_, C^5^H_2_, C^6^H_2_, C^7^H_2_, C^8^H_2_), 0.84 (3H, t, *J* = 6.7 Hz, CH_3_). ^13^C-NMR (DMSO-*d*_6_): δ 165.39, 158.34, 136.81, 131.65 (q, *J* = 31.8 Hz), 128.66, 125.47 (q, *J* = 3.7 Hz), 124.08 (q, *J* = 272.5 Hz), 39.40, 31.48, 30.03, 29.20, 29.02, 28.86, 26.49, 22.28, 14.12. Anal. Calcd for C_18_H_26_F_3_N_3_O_2_ (373.41): C, 57.90; H, 7.02; N, 11.25. Found: C, 58.07; H, 6.95; N, 11.31.

*N-Decyl-2-[4-(trifluoromethyl)benzoyl]hydrazine-1-carboxamide* (**2j**). White solid; yield 90 % (method B); mp 204–205 °C. IR (ATR): 3301, 2921, 2854, 1667, 1638, 1584, 1548, 1471, 1327, 1314, 1529, 1172, 1122, 1073, 1015, 857, 719, 695, 645, 630 cm^−1^. ^1^H-NMR (DMSO-*d*_6_): δ 10.31 (1H, s, NH), 8.07 (2H, d, *J* = 8.1 Hz, H2, H6), 7.88–7.83 (3H, m, NH, H3, H5), 6.50 (1H, t, *J* = 5.8 Hz, NH-alkyl), 3.00 (2H, q, *J* = 6.6 Hz, N-CH_2_), 1.37 (2H, p, *J* = 6.8 Hz, C^2^H_2_), 1.29–1.18 (14H, m, C^3^H_2_, C^4^H_2_, C^5^H_2_, C^6^H_2_, C^7^H_2_, C^8^H_2_, C^9^H_2_), 0.84 (3H, t, *J* = 6.9 Hz, CH_3_). ^13^C-NMR (DMSO-*d*_6_): δ 165.34, 158.29, 136.79, 131.62 (q, *J* = 32.1 Hz), 128.62, 125.43 (q, *J* = 3.8 Hz), 124.05 (q, *J* = 272.5 Hz), 39.37, 31.45, 30.00, 29.21, 29.13, 28.98, 28.87, 26.46, 22.24, 14.07. Anal. Calcd for C_19_H_28_F_3_N_3_O_2_ (387.44): C, 58.90; H, 7.28; N, 10.85. Found: C, 58.82; H, 6.99; N, 11.00.

*2-[4-(Trifluoromethyl)benzoyl]-N-undecylhydrazine-1-carboxamide* (**2k**). White solid; yield 98 % (method A); mp 201.5–203 °C. IR (ATR): 3304, 3070, 2955, 2920, 2853, 1667, 1638, 1583, 1545, 1471, 1377, 1343, 1328, 1315, 1259, 1170, 1122, 1073, 1015, 857, 718, 696, 629 cm^−1^. ^1^H-NMR (DMSO-*d*_6_): δ 10.30 (1H, s, NH), 8.06 (2H, d, *J* = 8.0 Hz, H2, H6), 7.88–7.83 (3H, m, NH, H3, H5), 6.51 (1H, t, *J* = 5.9 Hz, NH-alkyl), 2.99 (2H, q, *J* = 6.6 Hz, N-CH_2_), 1.38 (2H, p, *J* = 6.8 Hz, C^2^H_2_), 1.29–1.18 (16H, m, C^3^H_2_, C^4^H_2_, C^5^H_2_, C^6^H_2_, C^7^H_2_, C^8^H_2_, C^9^H_2_, C^10^H_2_), 0.83 (3H, t, *J* = 6.7 Hz, CH_3_). ^13^C-NMR (DMSO-*d*_6_): δ 165.50, 158.41, 136.87, 131.69 (q, *J* = 32.1 Hz), 128.72, 125.56 (q, *J* = 3.9 Hz), 124.04 (q, *J* = 272.5 Hz), 39.39, 31.53, 30.07, 29.29, 29.27, 29.25, 29.06, 28.95, 26.53, 22.33, 14.19. Anal. Calcd for C_20_H_30_F_3_N_3_O_2_ (401.47): C, 59.83; H, 7.53; N, 10.47. Found: C, 59.94; H, 7.74; N, 10.65.

*N-Dodecyl-2-[4-(trifluoromethyl)benzoyl]hydrazine-1-carboxamide* (**2l**). White solid; yield 99 % (method A); mp 197.5–200 °C. IR (ATR): 3306, 3071, 2956, 2917, 2851, 1667, 1640, 1584, 1545, 1471, 1441, 1327, 1315, 1254, 1172, 1122, 1073, 1015, 857, 718, 696, 628 cm^−1^. ^1^H-NMR (DMSO-*d*_6_): δ 10.31 (1H, s, NH), 8.07 (2H, d, *J* = 8.0 Hz, H2, H6), 7.88–7.84 (3H, m, NH, H3, H5), 6.50 (1H, t, *J* = 5.6 Hz, NH-alkyl), 3.00 (2H, q, *J* = 6.6 Hz, N-CH_2_), 1.37 (2H, p, *J* = 6.8 Hz, C^2^H_2_), 1.30–1.17 (18H, m, C^3^H_2_, C^4^H_2_, C^5^H_2_, C^6^H_2_, C^7^H_2_, C^8^H_2_, C^9^H_2_, C^10^H_2_, C^11^H_2_), 0.84 (3H, t, *J* = 6.7 Hz, CH_3_). ^13^C-NMR (DMSO-*d*_6_): δ 165.38, 158.32, 136.82, 131.64 (q, *J* = 32.0 Hz), 128.65, 125.47 (q, *J* = 3.9 Hz), 124.08 (q, *J* = 272.6 Hz), 39.34, 31.47, 30.00, 29.25, 29.24, 29.20, 29.13, 29.01, 28.89, 26.48, 22.27, 14.12. Anal. Calcd for C_21_H_32_F_3_N_3_O_2_ (415.49): C, 60.70; H, 7.76; N, 10.11. Found: C, 60.69; H, 7.74; N, 10.27.

*N-Tridecyl-2-[4-(trifluoromethyl)benzoyl]hydrazine-1-carboxamide* (**2m**). White solid; yield 91 % (method B); mp 199–200 °C. IR (ATR): 3302, 2918, 2851, 1667, 1639, 1583, 1546, 1471, 1328, 1315, 1263, 1171, 1122, 1073, 1015, 857, 718, 696, 640, 622 cm^−1^. ^1^H-NMR (DMSO-*d*_6_): δ 10.30 (1H, s, NH), 8.06 (2H, d, *J* = 8.1 Hz, H2, H6), 7.89–7.84 (3H, m, NH, H3, H5), 6.50 (1H, t, *J* = 5.6 Hz, NH-alkyl), 3.00 (2H, q, *J* = 6.6 Hz, N-CH_2_), 1.42–1.15 (22H, m, C^2^H_2_, C^3^H_2_, C^4^H_2_, C^5^H_2_, C^6^H_2_, C^7^H_2_, C^8^H_2_, C^9^H_2_, C^10^H_2_, C^11^H_2_, C^12^H_2_), 0.84 (3H, t, *J* = 6.8 Hz, CH_3_). ^13^C-NMR (DMSO-*d*_6_): δ 165.35, 158.29, 136.80, 131.61 (q, *J* = 32.0 Hz), 128.63, 125.46 (q, *J* = 3.9 Hz), 124.05 (q, *J* = 272.6 Hz), 39.37, 31.45, 30.00, 29.28–29.11 (5C, overlapping), 28.98, 28.86, 26.45, 22.25, 14.10. Anal. Calcd for C_22_H_34_F_3_N_3_O_2_ (429.52): C, 61.52; H, 7.98; N, 9.78. Found: C, 61.44; H, 7.86; N, 10.01.

*N-Tetradecyl-2-[4-(trifluoromethyl)benzoyl]hydrazine-1-carboxamide* (**2n**). White solid; yield 97 % (method A); mp 193.5–195.5 °C. IR (ATR): 3302, 3918, 2851, 1667, 1639, 1584, 1546, 1471, 1379, 1328, 1314, 1258, 1172, 1122, 1073, 1015, 857, 719, 695, 637, 628 cm^−1^. ^1^H-NMR (DMSO-*d*_6_): δ 10.31 (1H, s, NH), 8.07 (2H, d, *J* = 8.1 Hz, H2, H6), 7.88–7.83 (3H, m, NH, H3, H5), 6.50 (1H, t, *J* = 5.6 Hz, NH-alkyl), 3.00 (2H, q, *J* = 6.7 Hz, N-CH_2_), 1.42–1.13 (24H, m, C^2^H_2_, C^3^H_2_, C^4^H_2_, C^5^H_2_, C^6^H_2_, C^7^H_2_, C^8^H_2_, C^9^H_2_, C^10^H_2_, C^11^H_2_, C^12^H_2_, C^13^H_2_), 0.84 (3H, t, *J* = 6.7 Hz, CH_3_). ^13^C-NMR (DMSO-*d*_6_): δ 165.35, 158.29, 136.80, 131.62 (q, *J* = 32.0 Hz), 128.63, 125.48 (q, *J* = 3.9 Hz), 124.06 (q, *J* = 272.6 Hz), 39.37, 31.45, 30.01, 29.29-29.12 (6C, overlapping), 28.98, 28.86, 26.46, 22.25, 14.11. Anal. Calcd for C_23_H_36_F_3_N_3_O_2_ (443.55): C, 62.28; H, 8.18; N, 9.47. Found: C, 62.39; H, 8.30; N, 9.54.

*N-Pentadecyl-2-[4-(trifluoromethyl)benzoyl]hydrazine-1-carboxamide* (**2o**). White solid; yield 78 % (method B); mp 178.5–180.5 °C. IR (ATR): 3308, 2954, 2919, 2850, 1667, 1638, 1582, 1554, 1471, 1329, 1315, 1259, 1170, 1122, 1073, 1015, 857, 719, 696, 644, 622 cm^−1^. ^1^H-NMR (DMSO-*d*_6_): δ 10.30 (1H, s, NH), 8.07 (2H, d, *J* = 8.0 Hz, H2, H6), 7.88–7.84 (3H, m, NH, H3, H5), 6.51 (1H, t, *J* = 5.7 Hz, NH-alkyl), 3.01 (2H, q, *J* = 6.6 Hz, N-CH_2_), 1.42–1.13 (26H, m, C^2^H_2_, C^3^H_2_, C^4^H_2_, C^5^H_2_, C^6^H_2_, C^7^H_2_, C^8^H_2_, C^9^H_2_, C^10^H_2_, C^11^H_2_, C^12^H_2_, C^13^H_2_, C^14^H_2_), 0.84 (3H, t, *J* = 6.8 Hz, CH_3_). ^13^C-NMR (DMSO-d_6_): δ 165.37, 158.30, 136.79, 131.64 (q, *J* = 32.0 Hz), 128.63, 125.46 (q, *J* = 3.8 Hz), 124.07 (q, *J* = 272.6 Hz), 39.37, 31.44, 30.00, 29.29-29.11 (7C, overlapping), 28.98, 28.86, 26.44, 22.24, 14.09. Anal. Calcd for C_24_H_38_F_3_N_3_O_2_ (457.57): C, 63.00; H, 8.37; N, 9.18. Found: C, 62.78; H, 8.44; N, 9.09.

*N-Hexadecyl-2-[4-(trifluoromethyl)benzoyl]hydrazine-1-carboxamide* (**2p**). White solid; yield 99 % (method A); mp 191.5–193.5 °C. IR (ATR): 33308, 2955, 2916, 2850, 1667, 1639, 1585, 1544, 1471, 1379, 1328, 1315, 1263, 1172, 1122, 1073, 1015, 857, 718, 696, 647, 638, 627 cm^−1^. ^1^H-NMR (DMSO-*d*_6_): δ 10.31 (1H, s, NH), 8.07 (2H, d, *J* = 8.0 Hz, H2, H6), 7.89–7.85 (3H, m, NH, H3, H5), 6.50 (1H, t, *J* = 5.7 Hz, NH-alkyl), 3.00 (2H, q, *J* = 6.6 Hz, N-CH_2_), 1.41–1.16 (28H, m, C^2^H_2_, C^3^H_2_, C^4^H_2_, C^5^H_2_, C^6^H_2_, C^7^H_2_, C^8^H_2_, C^9^H_2_, C^10^H_2_, C^11^H_2_, C^12^H_2_, C^13^H_2_, C^14^H_2_, C^15^H_2_), 0.85 (3H, t, *J* = 6.7 Hz, CH_3_). ^13^C-NMR (DMSO-*d*_6_): δ 165.35, 158.31, 136.78, 131.65 (q, *J* = 32.0 Hz), 128.64, 125.46 (q, *J* = 3.9 Hz), 124.08 (q, *J* = 272.6 Hz), 39.37, 31.44, 30.00, 29.29–29.10 (8C, overlapping), 28.97, 28.86, 26.44, 22.23, 14.10. Anal. Calcd for C_25_H_40_F_3_N_3_O_2_ (471.60): C, 63.67; H, 8.55; N, 8.91. Found: C, 63.73; H, 8.69; N, 8.77.

*N-Octadecyl-2-[4-(trifluoromethyl)benzoyl]hydrazine-1-carboxamide* (**2q**). White solid; yield 98 % (method A); mp 193.5–196 °C. IR (ATR): 3303, 2191, 2850, 1666, 1639, 1586, 1548, 1470, 1329, 1315, 1256, 1172, 1122, 1074, 1015, 857, 719, 695, 648, 640, 630 cm^−1^. ^1^H-NMR (DMSO-*d*_6_): δ 10.30 (1H, s, NH), 8.07 (2H, d, *J* = 8.4 Hz, H2, H6), 7.88–7.84 (3H, m, NH, H3, H5), 6.49 (1H, t, *J* = 5.9 Hz, NH-alkyl), 3.00 (2H, q, *J* = 7.0 Hz, N-CH_2_), 1.41–1.12 (32H, m, C^2^H_2_, C^3^H_2_, C^4^H_2_, C^5^H_2_, C^6^H_2_, C^7^H_2_, C^8^H_2_, C^9^H_2_, C^10^H_2_, C^11^H_2_, C^12^H_2_, C^13^H_2_, C^14^H_2_, C^15^H_2_, C^16^H_2_, C^17^H_2_), 0.85 (3H, t, *J* = 6.9 Hz, CH_3_). ^13^C-NMR (DMF, 80 °C): δ 165.95, 158.48, 137.48, 132.13 (q, *J* = 33.9 Hz), 128.44, 125.29 (q, *J* = 3.8 Hz), 124.10 (q, *J* = 272.5 Hz), 39.91, 31.63, 30.14, 29.38, 29.31, 29.14, 28.99, 28.90 (overlapping signals, partly overlapping with solvent signals), 26.71, 22.29, 13.38. Anal. Calcd for C_27_H_44_F_3_N_3_O_2_ (499.65): C, 64.90; H, 8.88; N, 8.41. Found: C, 65.04; H, 8.99; N, 8.30.

*N′*-*Hexanoyl-4-(trifluoromethyl)benzohydrazide* (**3a**). White solid; yield 79 % (method A); mp 188–190 °C. IR (ATR): 3203, 2953, 2874, 1600, 1572, 1516, 1475, 1423, 1406, 1324, 1223, 1165, 1126, 1109, 1067, 1016, 860, 726, 690, 648, 628 cm^−1^. ^1^H-NMR (DMSO-*d*_6_): δ 10.52 (1H, s, NH), 9.91 (1H, s, NH), 8.05 (2H, d, *J* = 8.1 Hz, H2, H6), 7.87 (2H, d, *J* = 8.1 Hz, H3, H5), 2.18 (2H, t, *J* = 7.4 Hz, CO-CH_2_), 1.55 (2H, p, *J* = 7.3 Hz, C^3^H_2_), 1.33–1.25 (4H, m, C^4^H_2_, C^5^H_2_), 0.87 (3H, t, *J* = 7.3 Hz, CH_3_). ^13^C-NMR (DMSO-*d*_6_): δ 171.71, 164.53, 136.53, 131.76 (q, *J* = 31.8 Hz), 128.52, 125.64 (q, *J* = 3.9 Hz), 124.04 (q, *J* = 272.3 Hz), 33.39, 30.93, 24.91, 22.03, 14.02. Anal. Calcd for C_14_H_17_F_3_N_2_O_2_ (302.29): C, 55.62; H, 5.67; N, 9.27. Found: C, 55.73; H, 5.88; N, 9.34.

*N′*-*Palmitoyl-4-(trifluoromethyl)benzohydrazide* (**3b**). White solid; yield 97 % (method B); mp 152–153.5 °C. IR (ATR): 3195, 2952, 2916, 2848, 1672, 1603, 1571, 1516, 1480, 1465, 1413, 1329, 1226, 1211, 1165, 1125, 1108, 1069, 1016, 873, 859, 767, 723, 693, 651, 627 cm^−1^. ^1^H-NMR (DMSO-*d*_6_): δ 10.50 (1H, s, NH), 9.89 (1H, s, NH), 8.05 (2H, d, *J* = 8.1 Hz, H2, H6), 7.88 (2H, d, *J* = 8.1 Hz, H3, H5), 2.17 (2H, t, *J* = 7.3 Hz, CO-CH_2_), 1.54 (2H, p, *J* = 7.3 Hz, C^3^H_2_), 1.33–1.14 (24H, m, C^4^H_2_, C^5^H_2_, C^6^H_2_, C^7^H_2_, C^8^H_2_, C^9^H_2_, C^10^H_2_, C^11^H_2_, C^12^H_2_, C^13^H_2_, C^14^H_2_, C^15^H_2_), 0.84 (3H, t, *J* = 7.3 Hz, CH_3_). ^13^C-NMR (DMF): δ 171.73, 164.65, 136.93, 132.09 (q, *J* = 32.2 Hz), 128.43, 125.50 (q, *J* = 3.7 Hz), 124.18 (q, *J* = 272.3 Hz), 33.54, 31.68, 29.48, 29.47, 26.45, 29.38, 29.22, 29.14, 29.00, 28.96 (overlapping signals, partly overlapping with solvent signals), 25.41, 22.39, 13.56. Anal. Calcd for C_24_H_37_F_3_N_2_O_2_ (442.56): C, 65.13; H, 8.43; N, 6.33. Found: C, 65.31; H, 8.25; N, 6.39.

*N*-*Hexyl-5-[4-(trifluoromethyl)phenyl]-1,3,4-oxadiazol-2-amine* (**4**). White solid; yield 82%; mp 91–92.5 °C. IR (ATR): 3202, 2954, 2874, 1599, 1572, 1517, 1475, 1323, 1223, 1170, 1125, 1109, 1066, 1015, 860, 726, 690, 649, 627 cm^−1^. ^1^H-NMR (DMSO-*d*_6_): δ 7.98 (2H, d, *J* = 8.2 Hz, H2, H6), 7.92 (1H, t, *J* = 5.7 Hz, NH), 7.87 (2H, d, *J* = 8.3 Hz, H3, H5), 3.24 (2H, td, *J* = 7.1, 5.6 Hz, N-CH_2_), 1.56 (2H, p, *J* = 7.1 Hz, C^2^H_2_), 1.36–1.23 (6H, m, C^3^H_2_, C^4^H_2_, C^5^H_2_), 0.85 (3H, t, *J* = 7.2 Hz, CH_3_). ^13^C-NMR (DMSO-*d*_6_): δ 164.14, 156.54, 130.15 (q, *J* = 32.0 Hz), 128.91, 126.35 (q, *J* = 3.8 Hz), 125.82, 124.03 (q, *J* = 272.2 Hz), 42.73, 31.04, 28.85, 26.00, 22.16, 13.99. Anal. Calcd for C_15_H_18_F_3_N_3_O (313.32): C, 57.50; H, 5.79; N, 13.41. Found: C, 57.59; H, 5.97; N, 13.59.

### 3.3. Determination of AChE and BuChE Inhibition

IC_50_ values were determined using the spectrophotometric Ellman’s method modified according to Zdražilová et al. [16], which is a simple, rapid and direct method to determine the SH and -S-S- group content. This method is widely used for the screening of the efficiency of cholinesterases inhibitors. The enzymatic activity is measured indirectly by quantifying the concentration of the 5-thio-2-nitrobenzoic acid ion formed in the reaction between the thiol reagent 5,5′-dithio-bis(2-nitrobenzoic acid) and thiocholine, a product of acetylthiocholine substrate hydrolysis by cholinesterases [20].

The enzyme activity in final reaction mixture (2000 µL) was 0.2 U/mL, concentration of acetylthiocholine (or butyrylthiocholine) 40 µM and concentration of 5,5′-dithio-bis(2-nitrobenzoic acid) 0.1 mM for all reactions. The tested compounds were dissolved in DMSO and then diluted in demineralized water to the concentration of 1 mM. For all the tested compounds and a standard carbamate rivastigmine five different concentrations of inhibitor in final reaction mixture were used. All measurements were carried in triplicates and the average values of reaction rate (v_0_-uninhibited reaction, v_i_-inhibited reaction) were used for construction of the dependence *v_0_*/*v_i_* vs. concentration of inhibitor. From obtained equation of regression curve, IC_50_ values were calculated.

Acetylcholinesterase used was obtained from electric eel (*Electrophorus electricus* L.; *Ee*AChE) and butyrylcholinesterase from equine serum (EqBuChE). Rivastigmine was used as a reference cholinesterase inhibitor. All the enzymes, substrates and rivastigmine were purchased from Sigma-Aldrich (Prague, Czech Republic).

### 3.4. Molecular Docking

Crystallographic structures of human AChE and human BChE were obtained from Protein Data Bank (www.rcsb.org; pdb codes 4PQE and 1POI, respectively). The 3D structures of ligands were prepared in Chem3D Pro 19.1 (ChemBioOffice 2019 Package, CambridgeSoft, Cambridge, MA, USA). In the preparation process, all water molecules were removed from the enzymes and structures of enzymes and ligands were optimized using UCSF Chimera software package (Amber force field) [21]. Docking was performed using Autodock Vina [22] and Autodock 4.2 [23] (a Lamarckian genetic algorithm was used). The 3D affinity grid box was designed to include the full active and peripheral site of AChE and BChE. The number of grid points in the x-, y- and z-axes was 20, 20 and 20 with grid points separated by 1 Å (Autodock Vina) and 40, 40 and 40 with grid points separated by 0.4 Å (Autodock 4). The graphic visualisations of the ligand-enzyme interactions were prepared in PyMOL (The PyMOL Molecular Graphics System, Version 1.5 Schrödinger, LLC, New York, NY, USA).

### 3.5. Antimycobacterial Activity

Derivatives of **1** were screened in vitro for their potential antimycobacterial activity against *M. tuberculosis* 331/88 (H_37_Rv; dilution of this strain was 10^−3^), *Mycobacterium avium* 330/88 (resistant to isoniazid, rifampicin, ofloxacin and ethambutol; dilution 10^−5^) and two strains of *M. kansasii*: 235/80 (dilution 10^−4^) and the clinically isolated *M. kansasii* strain 6509/96 (dilution 10^−4^) according to ref. [24]. The collection strains were obtained from Czech National Collection of Type Cultures (Prague, Czech Republic). The following concentrations were used: 1000, 500, 250, 125, 62.5, 32, 16, 8, 4, 2, and 1 μM. MIC (reported in μM) was the lowest concentration at which the complete inhibition of mycobacterial growth occurred. Isoniazid (INH) as a first-line oral antimycobacterial drug and the synthetic precursor 4-(trifluoromethyl)benzohydrazide (**1**) were used as reference compounds.

### 3.6. Cytostatic Activity Evaluation

HepG2, human hepatocellular carcinoma cells (ATCC-HB8065) and MonoMac-6 cells (CC124; Deutsche Sammlung von Mikroorganismen and Zellkulturen GmbH, Braunschweig, Germany) were cultured in RPMI-1640 medium containing 10% foetal calf serum (FCS), 2 mM L-glutamine, 160 µg/mL gentamycin at 37 °C, 5% CO_2_ in water-saturated atmosphere and grown to confluence and were plated into 96-well plate with initial cell number of 5.0–10.0 × 10^3^ per well. After 24 h incubation at 37 °C, cells were treated with the compounds in 200 μL final volume containing 1.0% (*v*/*v*) DMSO. Cells were incubated with the compounds at 0.0128–100 µM concentration range for overnight. Control cells were treated with serum free medium (RPMI-1640) only or with DMSO (*c* = 1.0% *v*/*v*) at 37 °C for overnight. After incubation, the cells were washed twice with serum free medium (RPMI-1640). Cells were cultured for a further 72 h in serum containing medium.

The cell viability was determined by 3-(4,5-dimethylthiazol-2-yl)-2,5-diphenyltetrazolium bromide (MTT) assay. The solution of MTT (45 μL, 2 mg/mL) was added to each well, which was reduced to insoluble violet formazan dye crystals within the living cells. After 3.5 h of incubation at 37 °C the cells were centrifuged for 5 min (2000 rpm) and supernatant was removed. The obtained formazan crystals were dissolved in 100 μL of DMSO and the optical density (OD) of the samples was measured at *λ* = 540 and 620 nm, respectively, employing an ELISA reader (iEMS Reader, Labsystems, Helsinki, Finland). OD620 values were subtracted from OD540 values and the percent of cytostasis was calculated using the following equation:

Cytostatic effect (%) = [1 − (OD_treated_/OD_control_)] × 100

where OD_treated_ and OD_control_ correspond to the optical densities of the treated and the control cells, respectively). For each compound, at least two independent experiments were carried out with four parallel measurements.

The 50% inhibitory concentration (IC_50_) values were determined from the dose-response curves. The curves were defined using Microcal^TM^ Origin1 version 7.6 software (OriginLab, Northampton, MA, USA). Cytostasis (%) was plotted as a function of concentration, fitted to a sigmoidal curve and, based on this curve, the half maximal inhibitory concentration (IC_50_) value was determined representing the concentration of a compound required for 50% inhibition in vitro.

## 4. Conclusions

Based on the isostere concept, we designed and prepared a series of *N*-alkyl-2-[4-(trifluoromethyl)benzoyl]hydrazine-1-carboxamides using three synthetic procedures. Although they were proposed as possible isoniazid analogues, their antimycobacterial activity was predominantly low. The most active *N*-hexyl-2-[4-(trifluoromethyl)benzoyl]hydrazine-1-carboxamide served as a lead structure for further structural optimization providing 1,2-diacylhydrazines and 1,3,4-oxadiazole. However, these modifications resulted in improved, but still mild antimycobacterial properties, again below expectation. Together with this antibacterial action, these compounds lack any cytostatic properties for eukaryotic cell lines despite the presence of a trifluoromethyl group.

The hydrazinecarboxamides were found to be dual inhibitors of both acetylcholinesterase and butyrylcholinesterase with IC_50_ values in the micromolar range. Almost all the derivatives inhibited AChE preferentially. *N*-Methyl, tridecyl and pentadecyl groups contributed to the most potent inhibition of AChE, while pentyl, hexyl and heptyl substituents led to an improved activity against BuChE. Molecular docking suggested the binding mode for the hydrazine-1-carboxamides, which are placed deep in the cavity in a close proximity to the active site triad.

In general, although designed as bioisosteres, new carboxamides did not behave pharmacologically in the same way as original isoniazid derivatives, in other words, they are not bioisosteres in the strict sense, but only isosteres. We identified several structure-activity relationships including the conclusion that the modification of the parent 4-(trifluoromethyl)benzohydrazide scaffold provided more active compounds with interesting biological properties for further structural optimization.

## Figures and Tables

**Table 1:** IC_50_ values for AChE and BuChE.

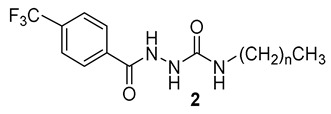				
Code	n	IC_50_ AChE (µM)	IC_50_ BuChE (µM)	Selectivity BuChE/AChE
**2a**	0	**31.23 ± 1.87**	84.16 ± 2.10	2.7
**2b**	1	56.32 ± 0.54	102.80 ± 2.75	1.8
**2c**	2	82.27 ± 3.31	112.70 ± 0.98	1.4
**2d**	3	38.60 ± 1.32	87.81 ± 7.96	2.3
**2e**	4	43.59 ± 0.41	**58.01 ± 0.78**	1.3
**2f**	5	49.16 ± 2.36	**72.47 ± 1.51**	1.5
**2g**	6	59.16 ± 2.38	**79.29 ± 1.53**	1.3
**2h**	7	76.97 ± 4.75	101.28 ± 0.99	1.3
**2i**	8	106.75 ± 1.73	82.24 ± 3.42	0.8
**2j**	9	49.47 ± 1.74	191.81 ± 6.83	3.9
**2k**	10	40.71 ± 0.37	179.12 ± 2.96	4.4
**2l**	11	45.25 ± 0.69	264.14 ± 0.22	5.8
**2m**	12	**28.90 ± 0.67**	277.48 ± 10.27	9.6
**2n**	13	38.74 ± 1.14	261.70 ± 17.20	6.8
**2o**	14	**27.04 ± 1.13**	233.18 ± 15.69	8.6
**2p**	15	68.63 ± 0.56	186.75 ± 13.24	2.7
**2q**	17	72.31 ± 2.01	145.72 ± 2.60	2.0
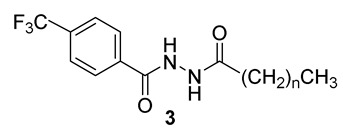				
**3a**	4	67.12 ± 3.05	109.98 ± 0.20	1.6
**3b**	14	91.87 ± 6.79	148.60 ± 0.36	1.6
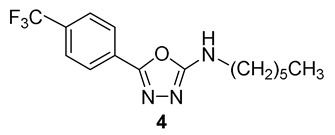				
**4**	-	71.32 ± 0.63	118.40 ± 1.46	1.7
**1**	-	69.37 ± 1.38	204.00 ± 2.69	2.9
Rivastigmine		56.10 ± 1.41	38.40 ± 1.97	0.7

AChE and BuChE inhibition are expressed as the mean ± SD (*n* = three independent experiments). The three lowest IC_50_ values for each enzyme are given in bold.
